# Real-time cell viability monitoring for high-throughput drug screening using tumor xenograft-derived cells

**DOI:** 10.1016/j.crmeth.2026.101464

**Published:** 2026-05-27

**Authors:** Elham Esmaeilishirazifard, Daniel Guerrero-Romero, Allan J.W. Lui, Abigail Shea, Long V. Nguyen, Maurizio Callari, Riccardo Masina, Kevin Tu, Richard Baird, Paul Edwards, Wendy Greenwood, Steve Fuller, Claire Crafter, Mandy Lawson, Alejandra Bruna, Oscar M. Rueda, Carlos Caldas

**Affiliations:** 1 Cancer Research UK Cambridge Institute, Li Ka Shing Centre, University of Cambridge, Cambridge CB2 0RE, UK; 2 Merck Life Science UK Limited, Gillingham SP8 4XT, UK; 3 Centre for Evolution and Cancer, Division of Molecular Pathology, The Institute of Cancer Research, Sutton, UK; 4 Princess Margaret Cancer Centre, University Health Network, Toronto, ON M5G 2M9, Canada; 5 Department of Medicine, and Department of Medical Biophysics, University of Toronto, Toronto, ON M5S 3H2, Canada; 6 Fondazione Michelangelo, Milan, Italy; 7 Department of Oncology, University of Cambridge, Cambridge, CB2 0AH, UK; 8 Varsity Pharmaceuticals Ltd, Cambridge, UK; 9 Bioscience, R&D Oncology, AstraZeneca, Cambridge, UK; 10 MRC-Biostatistics Unit, University of Cambridge, School of Clinical Medicine, Cambridge CB2 0SR, UK; 11 Department of Clinical Biochemistry and Institute of Metabolic Science, University of Cambridge, Cambridge, UK; 12 Department of Immunology and Cancer Research, Faculty of Medicine, Hebrew University of Jerusalem, Jerusalem, Israel

**Keywords:** patient-derived tumor xenografts, PDTXs, PDTX-derived tumor cells, PDTCs, real-time viability monitoring, high-throughput drug screening, breast cancer, pharmacodynamic profiling, *ex vivo* drug sensitivity, drug response kinetics, isotonic regression modeling, dynamic viability monitoring

## Abstract

Patient-derived tumor xenografts (PDTXs) recapitulate the molecular and phenotypic heterogeneity of human cancers, making them valuable pre-clinical models for cancer drug development. However, high-throughput drug screening (HTDS) using *ex vivo* short-term cultures of PDTX-derived tumor cells (PDTCs) is hindered by endpoint viability assays that provide only static measures of drug response. Here, we establish an optimized a screening platform by validating the RealTime-Glo (RTG) bioluminescent assay for dynamic, real-time measurements of PDTC viability. We further introduce an analytical metric to quantify drug responses independent of cell growth rate. Using this approach, we screened 67 compounds across 43 breast cancer PDTCs and revealed model-specific pharmacodynamic heterogeneity. Our PDTC-based HTDS pipeline improves assay robustness and offers an enhanced platform for leveraging patient-derived xenograft models in precision medicine.

## Highlights

• Real-time luminescent assay captures dynamic drug responses in PDTCs
• Continuous viability monitoring reveals early, late, and model-dependent drug responses
• RTG-IC50 metric decouples drug sensitivity from heterogeneous PDTC growth rates
• Sixty-seven compounds screened across 43 breast cancer PDTCs using CTC and RTG

## Motivation

High-throughput drug screens using PDTX-derived tumor cells (PDTCs) have been limited by viability assays that provide only static, endpoint measurements and require separate samples at each time point, preventing continuous observation of treatment effects. Moreover, intrinsic differences in tumor cell proliferation rates can skew endpoint readouts, making it hard to discern true drug sensitivity from growth-related variability. To address these gaps, we have implemented a non-lytic RealTime-Glo luminescent assay for real-time viability monitoring and introduced a metric that normalizes drug response independently of cell growth rate. This approach enables dynamic assessment of drug effects, overcoming key limitations of previous PDTC assays.

## Graphical abstract

**Figure:**
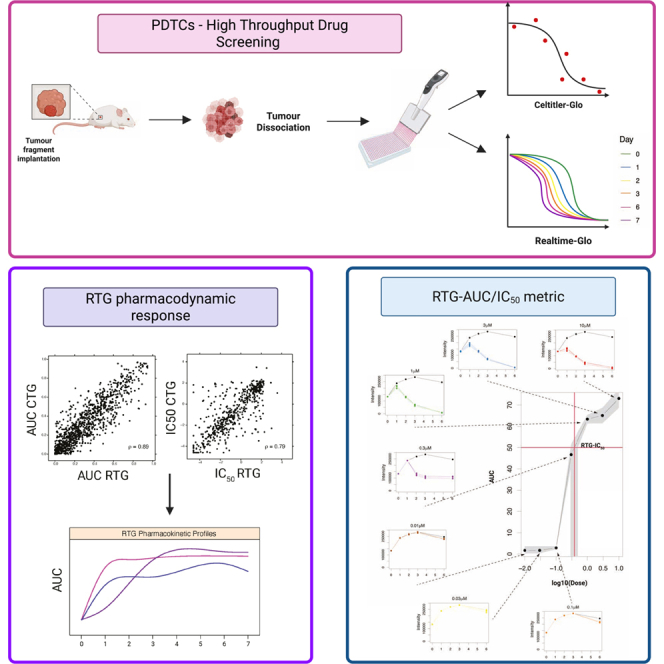

## Teaser

Esmaeilishirazifard et al. present a high-throughput drug screening platform that uses real-time bioluminescent monitoring to measure the viability of PDTX-derived tumor cells during drug treatment. The method reveals dynamic drug response patterns and introduces a growth rate-independent metric, improving the identification of effective therapies across diverse cancer models.

## Introduction

A major barrier to developing anticancer agents stems largely from the inadequacy of existing pre-clinical models in capturing the heterogeneity and molecular characteristics of human cancers.^1^ Patient-derived tumor xenografts (PDTX) have emerged as improved pre-clinical models that recapitulate the complex molecular, histological, and phenotypic heterogeneity of human tumors while providing renewable sources of malignant tissue that closely resemble the disease in patients.^2^^,^^3^^,^^4^^,^^5^^,^^6^^,^^7^ Compared to conventional cell lines and genetically engineered mouse models, PDTXs demonstrate higher predictive power for clinical drug responses, underscoring their potential to improve precision cancer medicine.^8^^,^^9^^,^^10^^,^^11^ Indeed, we recently demonstrated that drug responses in our breast cancer PDTXs closely mirror those observed in the corresponding patients, validating their clinical relevance.^11^ However, PDTX platforms for drug development have limited scalability, precluding their use in high-throughput drug screening (HTDS).

To enable broader drug testing, our group and others established short-term cultures of tumor cells derived from PDTXs, referred to as PDTCs.^3^ PDTCs retain the key molecular characteristic of the original tumors (the molecular “foot-print”) and enable *ex vivo* HTDS to identify biomarkers of drug response and resistance.^6^ Despite this advance, assessing drug responses in PDTC cultures remains challenging because it typically relies on endpoint viability assays, such as the ATP-based CellTiter-Glo 3D (CTG) assay.^9^^,^^12^^,^^13^^,^^14^ Endpoint assays require using separate cell samples for each time point, hindering the continuous tracking of drug effects over time. Moreover, inherent differences in PDTC growth rates (GRs)^3^ can confound endpoint measurements, making it difficult to distinguish true drug sensitivity from variations in proliferation.

The RealTime-Glo (RTG) is a non-lytic luminescent assay for continuous viability monitoring. It uses two components—a cell-permeant pro-substrate (RealTime-Glo MT Cell Viability Substrate) and a thermostable NanoLuc luciferase enzyme—present in the culture medium.^15^ In metabolically active cells, intracellular NAD(P)H-dependent oxidoreductases reduce the pro-substrate to the NanoLuc substrate (furimazine), which rapidly diffuses into the medium and is covered to light by NanoLuc, yielding a signal that, under recommended reagent excess, approaches a steady state proportional to the number of viable cells. Cells that lose reducing capacity cease to generate substrate, decreasing luminescence. In our PDTC experiments, the signal remains robust over multi-day intervals (typically ∼5–6 days before reagent depletion), enabling repeated reads from the same wells without lysis. We use these properties to quantify drug-response kinetics and benchmark them against traditional endpoint assays.^16^^,^^17^

For comparison, CTG quantifies ATP in a lytic, endpoint format. Because lysis precludes longitudinal reads from the same wells, CTG requires parallel plates or replicate wells for time courses, increasing between-well/plate variation. In this study, we benchmark RTG-derived growth and drug-response metrics against CTG endpoints acquired at matched time points.

Another strategy to account for differential cell proliferation in drug assays is the GR inhibition metric, which normalizes drug response measurements to the untreated GR of each model.^18^ However, standard GR calculations assume relatively consistent growth conditions and thus may not fully capture complex drug response scenarios, such as uneven or adaptative proliferation rates observed in PDTCs’ adaptation to *in vitro* conditions. Moreover, applying GR longitudinally would still require destructive sampling at each time point. In such cases, time-dependent GR metrics or additional modification may be necessary to accurately describe long-term drug response kinesics.

Here, we present a comprehensive HDTS pipeline for PDTCs by (1) implementing the RTG continuous viability assay to capture drug responses across both time and dose and (2) introducing an analytical metric that is robust to variability in growth kinetics across PDTCs. This metric normalizes drug responses independently of varying proliferation rates. We validate our approach across diverse patient-derived models and an extensive compound panel, highlighting complex pharmacodynamics patterns that conventional endpoint assays can miss.

## Results

### A validated platform of PDTCs from different cancer models for drug testing

**Figure 1:**
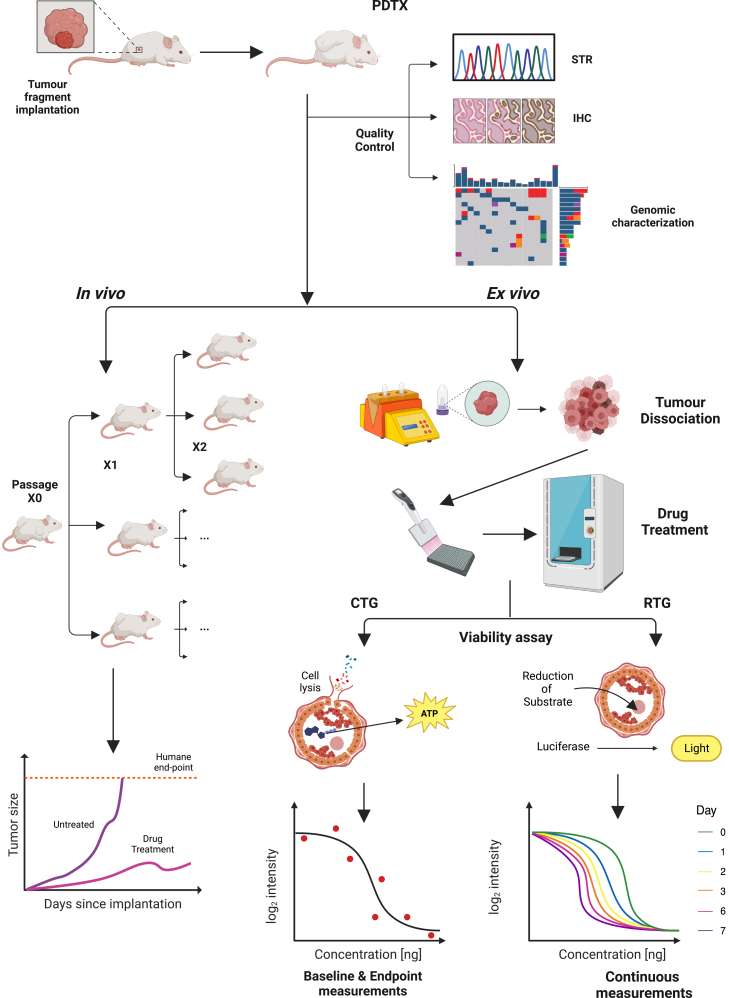
Workflow for HTDS using PDTCs PDTXs are propagated by the implantation of tumor fragments into NSG mice. At the humane endpoint, tumors are collected and subjected to quality control using STR profiling, IHC, and genomic characterization. PDTCs are generated by dissociating PDTX tissue and cultured *ex vivo*. Cells are dosed with drugs using an Echo Liquid Handler 550 and cell viability assayed daily for 7 days using RTG and/or after 7 days of treatment using CellTiter-Glo 3D.

We established a robust and reproducible drug screening platform using PDTXs and *ex vivo* short-term cultures PDTCs (Figure 1). Patient tumors spanning the major breast cancer subtypes were engrafted into immunodeficient NSH mice to generate PDTXs. After rigorous validation, a total of 42 breast cancer PDTXs were confirmed for drug testing, comprising 19 from triple-negative breast cancer (TNBC), 18 from estrogen receptor (ER)-positive/human epidermal growth factor receptor two HER2-negative (ER+/HER2-) breast cancer, and five from HER2+ breast cancer (two ER- and three ER+).

As illustrated in Figure 1, at the time of tumor harvest each PDTX was authenticated by short tandem repeats (STRs) genotyping to confirm its DNA profile matched that of the original patient tumor. We further ensured that each xenograft remained a human epithelial malignancy with no murine or non-epithelial overgrowth by performing immunohistochemistry (IHC) with a panel of markers (anti-mouse/human CD45, anti-human CD20, and anti-human AE1/AE3). These stringent quality control steps identified five aberrant xenografts—two human tumors of non-epithelial linage and three of mouse origin (Table S1)—which were excluded from further use. The final panel thus consisted of 42 fully authenticated human breast cancer PDTXs, providing a diverse foundation for PDTC-based drug screening.

All PDTC drug assays were performed with replicate testing to ensure consistent results. In addition, we characterized the genomic and transcriptomic profiles of each model, further confirming their authenticity and capturing their molecular diversity. Together, this validated set of breast cancer models and the rigorous verification workflow provide a solid platform for reproducible pre-clinical drug testing.

### Divergent growth rated between PDTCs and PDTXs

**Figure 2:**
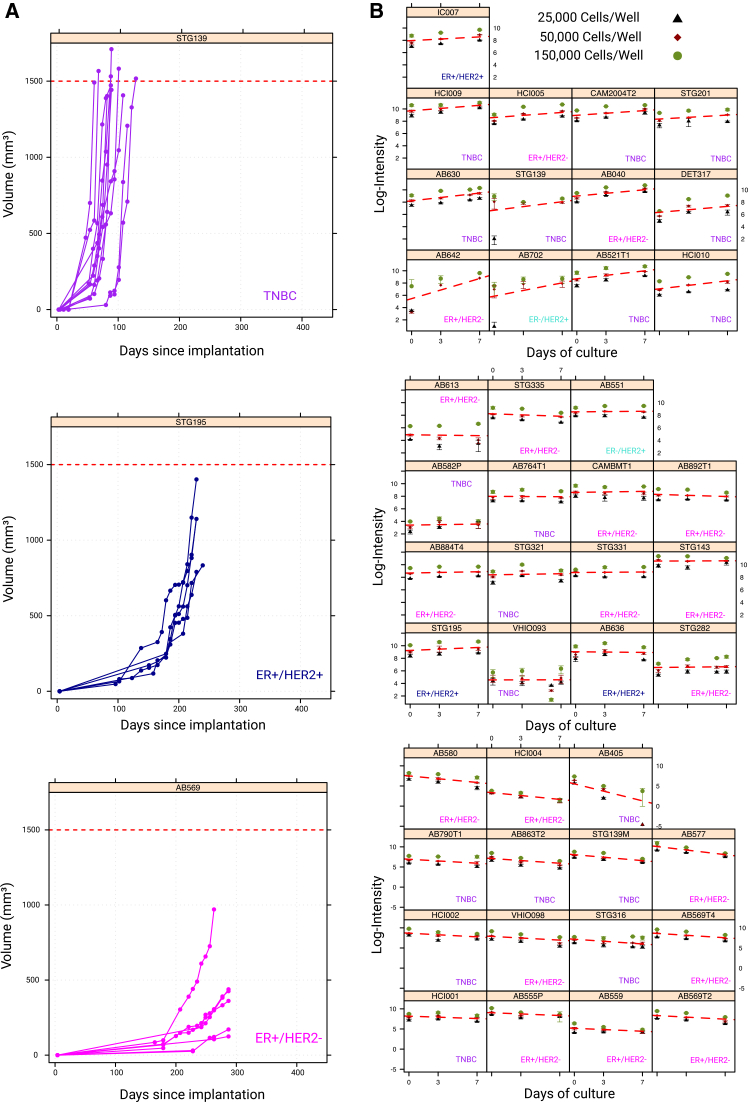
Growth kinetics of PDTX and PDTC models (A) Representative PDTX growth curves for models with fast (STG139, top), neutral (STG195, middle), and slow (AB569, bottom) GRs. Each line represents an individual replicate (i.e., mouse). The humane endpoint for PDTX tumor size is represented by the red dotted line. Hormone receptor and HER2 expression status are displayed. (B) PDTC growth at different seeding densities was measured by CTG assay for up to 7 days of culture and represented by log(luminescence intensity). Error bars, where shown, indicate variability in log-luminescence intensity among replicate wells for each seeding density at each time point. Models are separated into fast (top; mean GR > 0.08), neutral (middle; mean GR between −0.08 and 0.08) or slow-growing (bottom; mean GR < −0.08) groups, with hormone receptor and HER2 expression status displayed. Linear regression across all seeding densities was performed and represented by the red dashed line. Day 0 denotes one day after seeding for equivalence with PDTCs under treatment.

PDTXs display diverse *in vivo* growth characteristics, including varying engraftment periods and tumor growth phases. The engraftment period (i.e., lag-time) refers to the duration from tumor fragment implantation to measurable tumor growth, while the subsequent growth phase spans until the humane endpoint size (less than 1.5 cm^3^) is reached. Figure 2A illustrates representative growth trajectories for three breast cancer subtypes (TNBC, ER+/HER2+, and ER+/HER2-), emphasizing the inherent variability among models. These *in vivo* growth patterns of PDTXs will influence the downstream design of xenopatient trials but have only imperfect correlations with the *in vitro* GRs of their derived PDTCs.

PDTCs derived from 42 distinct PDTXs were therefore systematically analyzed and exhibited variable *in vitro* GRs. We assessed three seeding densities (25,000, 50,000, and 150,000 cells/well) and tested viability using the CellTiter-Glo 3D (CTG) assay^3^ at 0, 3, 7, 9, and 12 days (Figure 2B; Figure S1A). The observed PDTC model-specific *in vitro* GRs were independent of the seeding density (Figure 2B).

The application of linear regression approaches to the data revealed three distinct GR dynamics: negative GR, characterized by a reduction in viable cell count over time (*n* = 15 PDTCs); neutral GR, characterized by a stable, viable cell count over time (*n* = 15 PDTCs); and positive GR, characterized by increased viable cell count with time (*n* = 13 PDTCs) (Figure 2B).

Breast cancer subtype appeared to influence PDTC growth dynamics to some extent (Figure 2B; Figure S1B). For instance, among PDTCs exhibiting positive GRs 8/13 (61.5%) originated from TNBC PDTXs; however, TNBC-derived PDTCs also frequently displayed negative GRs 7/15 (47%). More predictably, only 3/19 PDTCs derived from ER+/HER2- PDTXs had positive GR, whereas 8/19 had neutral and 8/19 negative GRs, respectively. Interestingly, PDTCs derived from HER2+ PDTXs had either positive (2/5) or neutral (3/5) GRs, respectively.

Additionally, we observed substantial inter-well/inter-plate variability using the CTG assay, resulting in inconsistencies in assessing the growth dynamics of PDTCs, as illustrated in Figure S2A. This variability has potential implication for assay reliability in HTDS contexts, underscoring the need for alternative, more stable viability assessment methods.

### RTG to test ongoing cell viability and drug sensitivity in PDTCs

Given the limitations of the endpoint CTG assay—notably that it requires cell lysis and separate wells for each time point, which introduces variability in *in vitro* PDTC assays—we explored the RTG assay as an alternative for continuous cell viability monitoring.^15^ RTG allows repeated measurements in the same well without destroying cells, potentially providing more consistent and dynamic readouts of drug response. However, a known challenge is that RTG’s luminescent reagents become depleted after several days in culture.^15^ We therefore first tested whether increasing the concentration of RTG’s enzyme and substrate could extend signal duration in PDTC cultures (Figure S3). Using a set of representative drugs (the mTOR^19^ inhibitor AZD2014, the topoisomerase inhibitor epirubicin,^20^ and the BET bromodomain inhibitor JQ1^21^), we found that higher reagent levels did not improve luminescence longevity or assay performance beyond the manufacturer’s standard protocol (Figure S3).

**Figure 3:**
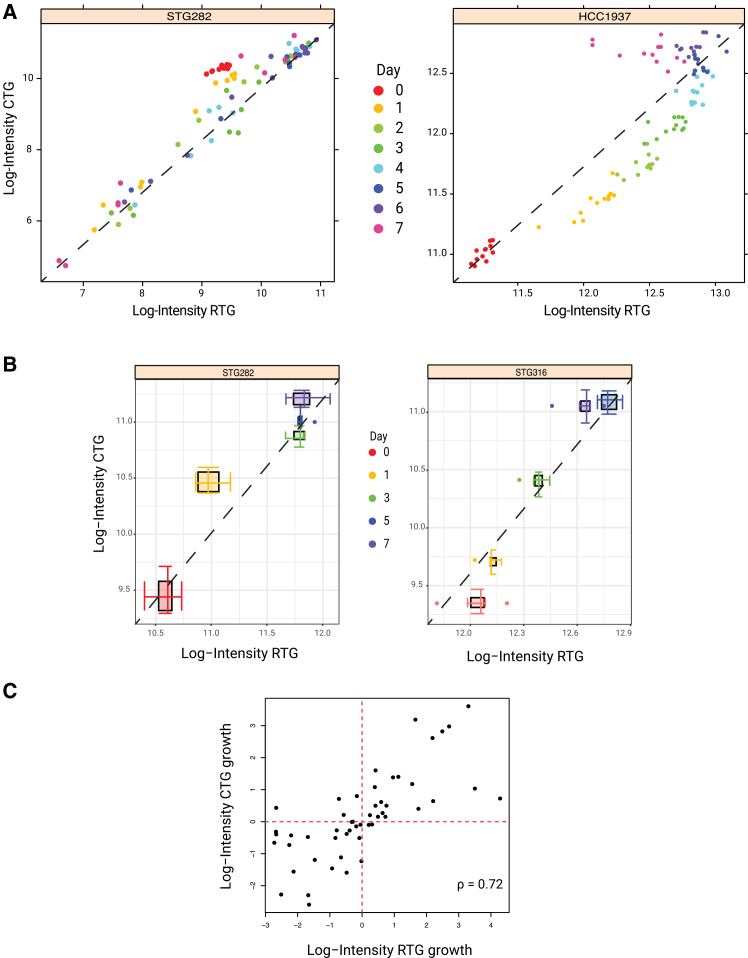
Correlation of CTG and RTG cell viability assays (A) STG282 PDTCs (left) or HCC1937 cells (right) were seeded into black 384-well plates, and cell viability quantified by CTG and RTG luminescence intensities from the same well after the indicated number of days of culture. Day 0 denotes one day after seeding to match with PDTCs time frame. (B) STG282 (left) or STG316 (right) PDTCs were seeded into black 384-well plates, and cell viability was quantified by CTG and RTG luminescence intensities from separate wells after the indicated number of days of culture and represented as two-dimensional boxplots. Horizontal and vertical error bars indicate variability among replicate wells in RTG and CTG log-intensities, respectively, at each time point. Day 0 denotes one day after seeding for equivalence with PDTCs under treatment. (C) Estimated GRs determined using CTG and RTG assays after 7 days of culture in 51 samples (28 different PDTX models and 3 cell lines). Pearson correlation = 0.72.

We next directly compared viability readouts from RTG and CTG under identical conditions to ensure RTG could reliably proxy for CTG. We performed a single-plate experiment (Figure S4A, experimental setting I) using both a breast cancer cell line (HCC1937, ER-/HER2-^22^) and a PDTC (STG282, an ER+/HER2-xenograft model). Cells were seeded at densities optimized from prior tests (200 cells/well for cell lines and 25,000 cells/well for PDTCs). Starting from day 0, we measured cell viability in designated wells every day for up to 7 days, first with RTG (which does not lyse the cells) and immediately thereafter with CTG on the same wells. This design allowed a direct one-to-one comparison of RTG vs. CTG signals in each well over time, albeit with each well being used only once (since CTG assay lyse the cells). Figure 3A shows the overlaid RTG and CTG measurements for the PDTC STG282 (left) and HCC1937 (right). Two assays demonstrated very close agreement in measured viability across the time course. Notably, the RTG and CTG curves remained nearly superimposable through day 5, and only began to diverge by days 6–7, when the RTG luminescent signal started to wane due to reagent depletion. These data confirmed that RTG can produce equivalent viability readouts to CTG in the same biological samples at least up to 5–6 days of culture, which is sufficient for most week drug assays.

To further examine the benefits of continuous same-well monitoring with RTG, we evaluated whether tracking the same cell population over time indeed reduces variability in growth measurements. We repeated the HCC1937 viability experiment using two setups: (1) the sequential RTG/CTG measurement as above (Figure S4A, experimental setting I) and (2) an RTG-only longitudinal measurement in which the same wells were read daily with RTG for 7 days without any CTG until the end (Figure S4A, experimental setting II). By comparing these approaches, we assessed the variance in GR estimates between day 3 and day 5. As expected, the continuous RTG tracking (single-well repeated measures) yielded the lowest variance in calculated growth change, indicating more consistent results than approaches requiring separate wells (Figures S4B and S4C). This finding encourages the rationale that using RTG to follow the same wells over time can mitigate inter-well variability inherent to endpoint assays.

To quantify this variance reduction, we performed a systematic comparison across all time points. Same-well RTG monitoring achieved a mean coefficient of variation (CV) of 7.0% (range: 3.5%–10.4%) which is acceptable for reproducibility. In contrast, different-well measurements showed markedly higher variability, with CTG and different-well RTG exhibiting mean CVs of 61.0% and 61.7%, respectively (Figure S4E). This 8.7-fold reduction in CV represents a 93.8% decrease in measurement variability. Statistical analysis using F-tests confirmed these differences were highly significant, with F-statistics ranging from 7.0 (day 1) to 116 (day 6), all *p* values < 0.001. The similarity between CTG and different-well RTG CVs shows that variance reduction derives from the same-well tracking methodology rather than assay chemistry.

In a second experimental approach, we evaluated RTG and CTG in a side-by-side manner using separate plates to mimic a practical high-throughput scenario. In this design (Figure S4A, setting III), two PDTCs (STG282 and STG316) were cultured in parallel: one plate was used for daily RTG readings, and a matching plate was used for CTG measurements at selected time points (days 0, 1, 3, 5, and 7). This setup reflects how one would typically perform longitudinal RTG monitoring without sacrificing the same wells, while using independent replicate wells for CTG at each endpoint. The results showed that both assays reported very similar cell viability trends over the week. The well-to-well variability in viability signals was comparable between RTG and CTG, and the values from the two assays were highly correlated at each time point (Figure 3B; Figure S4D). By day 7, we did observe the anticipated reduction in RTG signal intensity due to reagent exhaustion, but importantly, this did not significantly affect the overall correlation with CTG measurements. In other words, even though the luminescence from RTG was still in agreement with the CTG results, underscoring that RTG can reliably quantify cell growth through the entire assay period.

To ensure our observations were generalisable, we extended the RTG vs. CTG comparison to a large panel of independent cultures. We measured 7-day GRs (net viability changes) in 51 different cultures—comprising 28 distinct PDTCs and 3 cell lines—using RTG and CTG in separate plates as per second approach. The GR for each culture was calculated from the viability readings (see method details), and we then compared the values obtained by RTG to those from CTG. The two assays showed a strong concordance across this diverse set of models, yielding very similar growth estimates. In fact, the RTG-derived and CTG-derived GRs were highly correlated (Pearson’s ρ ≈ 0.72) as shown in Figure 3C. This high correlation across many drug screening experiments demonstrates that RTG is quantitatively equivalent to CTG in assessing cell proliferation and drug sensitivity in PDTCs.

### Comparing drug response metrics using RTG and CTG

Having confirmed that RTG and CTG yield similar baseline growth measurements (e.g., a 7-day GR correlation of 0.72), we next directly compared their performance in quantifying drug responses. In particular, we examined whether RTG—with its continuous, real-time readouts—can match the established CTG endpoint assay in measuring key drug-response parameters. We focused on two standard metrics: the area under the curve (AUC), which reflects cumulative drug efficacy, and the half-maximal inhibitory concentration (IC_50_), which reflects drug potency. To ensure a fair comparison, both assays were performed in parallel under identical conditions, and measurements were taken at the same endpoints. Because CTG provides only an endpoint readout while RTG continuously monitors cell viability, we selected two representative time points (day 3 and day 7) at which to compare metrics from the two platforms. This experimental design encompassed 67 anticancer compounds tested across 43 PDTCs, yielding a total of 1,117 drug-screening experiments—a sample size sufficient for robust statistical comparisons. For each PDTC-drug pair, we derived AUC and IC_50_ values from. The dose-response data of both assays (with IC_50_ values log10-transformed for analysis) at day 3 and day 7 (see method details). This approach allowed us to rigorously assess the sensitivity and reliability of the RTG assay relative to the gold-standard CTG in an HTDS context.

**Figure 4:**
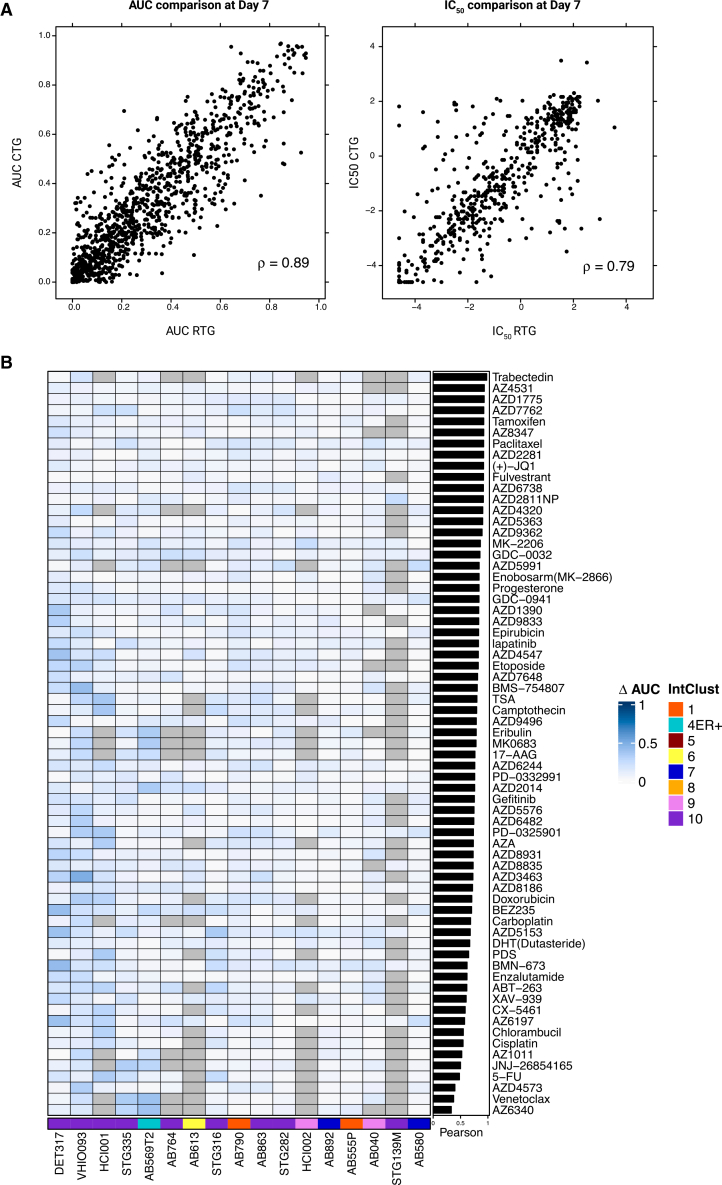
Comparison of CTG and RTG in PDTC high-throughput drug screening and RTG-associated pharmacokinetics (A) Comparison of area under the drug response curve (AUC) (left) and IC_50_ (right) obtained with CTG and RTG assays at day 7, for 67 drugs tested on 19 PDTC models for a total of 1,117 conditions. (B) Comparison of drug response profiling by endpoint and real-time cell viability assays. Heatmap displaying differences in AUC values between CTG and RTG for each drug-PDTC combination. Each column represents a PDTC model (with their respective IntClust subtype) and each row represents a drug. Cells are colored based on difference in AUC between CTG and RTG for a given drug-PDTC combination, with white indicating minimal difference and blue indicating a large difference.

The RTG and CTG assays proved to be highly concordant in their quantification of drug responses. As shown in Figure 4A, AUC values obtained from RTG on day 7 were strongly correlated with those from CTG (Pearson’s ρ ≈ 0.89), and the IC_50_ estimates from the two assays were likewise in close agreement (ρ ≈ 0.79 for log10 IC_50_). A similarly high correspondence was observed at the earlier 3-day endpoint (Figure S5A), with Pearson’s ρ ≈ 0.79 and ρ ≈ 0.82 for log10 IC_50_. These strong correlations indicate that RTG and CTG measure drug-induced viability changes almost interchangeably, despite the differing readout formats of the assays. In other words, RTG’s real-time luminescence output captures drug efficacy metrics that are equivalent to those from the well-established CTG endpoint assay. We further examined the agreement between assays by comparing the actual response values. A heatmap analysis of the difference in AUC (ΔAUC) for each drug-model combination revealed only minor discrepancies between CTG and RTG measurements at day 7 (lighter colors clustering around ΔAUC ≈ 0 in the heatmap), with no systematic bias favoring either assay (Figure 4B). This visual comparison underscores the high level of concordance across all 67 drugs and 43 PDTCs tested. Consistently, both assays ranked compounds’ effectiveness similarly across the diverse PDTC panel, and no outlier model or drug was identified where the two readouts diverged markedly.

**Figure 5:**
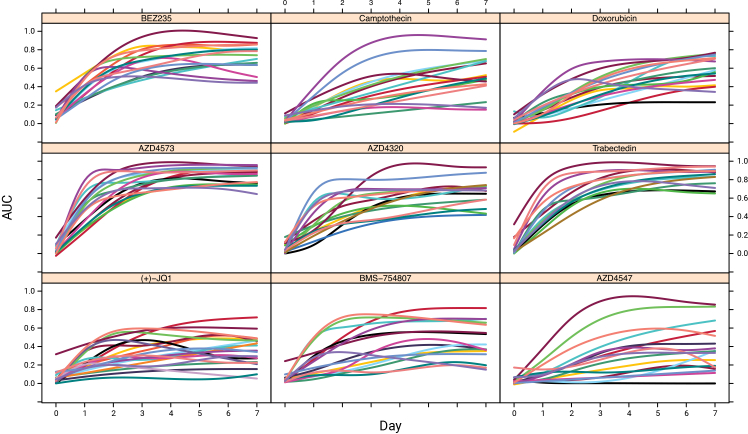
RTG-associated pharmacokinetics B-spline regression was performed on AUCs determined by continuous RTG luminescence measurements for up to 7 days of treatment with the indicated drug in up to 27 PDTC models. Each line represents a separate PDTC model. Representative plots of drugs reaching the AUC steady state at late, early or highly model-dependent time points are displayed on the top, middle, or bottom rows, respectively.

The potential added value of RTG lies on its ability to continuously monitor the *in vitro* growth of PDTCs. A B-spline regression model was fitted to the AUC values computed from the RTG readouts every day for 27 PDTC models treated with nine different drugs (Figure 5). This revealed that the dynamics of response in PDTCs to drugs fell into defined patterns: (1) late-response drugs, when AUCs reached a plateau on day 4 or later across models (Figure 5, top row); (2) early-response drugs, when AUC peaked within the first 2 days across models (Figure 5, middle row); and (3) model-dependent-response, when AUCs (i.e., GR inhibition) were highly variable across PDTC models (Figure 5, bottom row). The ability of RTG to capture dynamic drug responses across models, irrespective of their ability to grow in short-term culture, is illustrated in Figure S5B with three PDTC models with different GRs tested with a panel of 67 compounds.

### Development of a drug sensitivity metric using RTG

**Figure 6:**
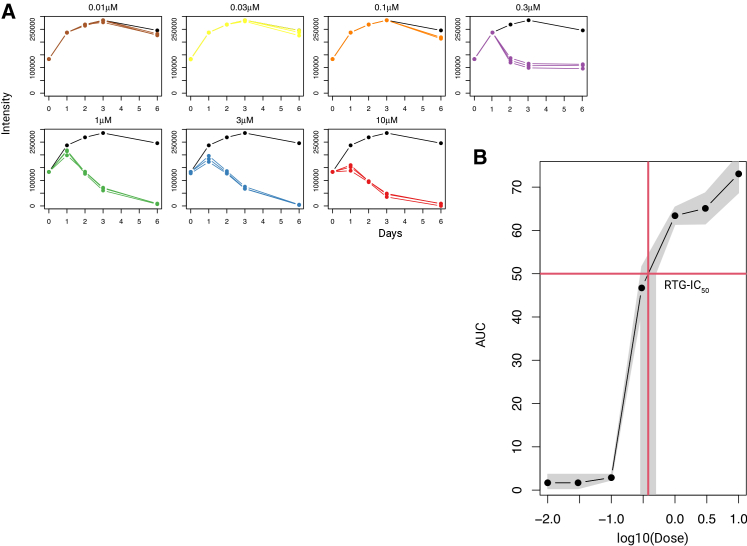
A proposed metric for RTG ex vivo screenings (A) Representation of cell growth dynamics via RTG viability assay for AB040-X1 PDTCs. A density of 25,000 cells/well was used for the assay. The PDTCs were treated with increasing concentrations of epirubicin, with each concentration tested in triplicate. The lines of varying colors in the plot illustrate the growth kinetics of AB040-X1 at distinct epirubicin concentrations across different time points, while the black line serves as the control, denoting cell growth under control vehicle treatment (DMSO). The graph accompanying each plot shows the area under the curve for each epirubicin concentration, effectively capturing the cumulative growth profile. (B) The data from (A) were used to compute the real-time growth inhibition concentration 50 (RTG-IC50), which is indicated bythe red vertical line, the gray shaded band represents the confidence interval from the isotonic regression model used to estimate the RTG-IC50.

Traditional drug responses metrics, which rely on single endpoint measurements (e.g., viability after a fixed time), are not well-suited for capturing the dynamic behaviors observed in RTG assays. To leverage the full temporal information from our longitudinal RTG viability data, we developed a drug sensitivity metric called RTG-IC_50_. This metric integrates multi-day growth response data into a single value that quantifies drug potency. Figure 6A illustrates this approach using PDTC AB040 treated with a range of epirubicin doses (0.01–10 μM). Higher drug concentrations lead to progressively greater growth inhibition over the 7-day assay, as evidenced by the reduced AUC at increased doses. Following a method similar to a previously described approach,^18^ we quantified the AUC of the RTG signal for each dose relative to the vehicle control (DMSO). This per-dose AUC represents the cumulative growth inhibition over time at that concentration.

We then combined the dose-response data across all concentrations to derive overall RTG-based metrics. In particular, we obtained an overall RTG-AUC for each drug and computed the RTG-IC_50_ by fitting an isotonic regression curve to the AUC values across doses (see algorithm 1; algorithm 2). Isotonic regression was chosen to enforce a monotonically decreasing dose-response relationship (i.e., no decrease in inhibition at higher doses) without assuming a specific sigmoidal model. Figure 6B shows the resulting monotonic dose-response curve for epirubicin in model AB040, from which RTG-IC_50_ is defined as the drug concentration causing 50% of maximal growth inhibition. The entire computation procedure—from replicate-wise AUC calculations to the final RTG-IC_50_ determination—is outlined in Figure S6A (flowchart of algorithm 1). A key advantage of this isotonic approach is its robustness in HTDS data: it can handle noisy or incomplete dose-response profiles that often arise in longitudinal assays.^23^ Notably, Fuji et al. (2015) demonstrated that isotonic regression outperforms classical sigmoidal curve-fitting under such suboptimal conditions, achieving ∼96% specificity in detecting true drug effects vs. ∼65% with the standard Hill equation model or even derived models (e.g., four-parameter log-logistic model or in short LL4). This evidence underpins the reliability of our RTG-IC_50_ method, as the isotonic estimator mitigates erratic data points and yields a stable dose-response from which to extract IC_50_ values.

Using this RTG-IC_50_ metric, we analyzed drug responses across multiple PDTCs. This RTG metric showed reasonable concordance with the conventional 7-day endpoint metrics from the CTG assay, though the correlation were moderate but significant (Pearson’s ρ ≈ 0.61 and 0.49 for log10 IC_50_, comparing RTG-derived vs. CTG-derived values). This level of correlation may indicate that while our RTG-based measures align with traditional readouts, they also capture unique aspects of the drug response. Indeed, by integrating responses over several days, the RTG-IC_50_ metric detects complex temporal dynamics of drug efficacy that a single endpoint measurement could miss. In practice, this means RTG-IC_50_ can distinguish different pharmacodynamic patterns—providing a more nuanced assessment of drug sensitivity than conventional endpoint IC_50_ values. Consequently, the RTG-IC_50_ metric offers a robust and information-rich tool for HTDS in PDTCs, leveraging real-time viability data to improve the characterization of drug response profiles.

## Discussion

Large HTDS using PDTCs requires robust experimental designs and analytical methods that mitigate technical variability and reliably capture biological complexity. To this end, our work addresses these challenges by establishing a validated pipeline integrating real-time continuous monitoring of PDTC viability and analytical metrics sensitive to treatment-induced growth dynamics. We confirmed RTG assay as a reliable alternative to the widely used CTG assay, demonstrating comparable performance while providing additional temporal insights into drug responses.

A primary advantage of RTG is its non-destructive, continuous viability monitoring, which significantly reduces inter-well and inter-plate variability inherent to endpoint assays like CTG. Indeed, our results demonstrate strong concordance between RTG and CTG metrics across multiple PDTCs treated with a broad panel of anticancer compounds, underscoring RTG’s reliability. Importantly, RTG’s longitudinal nature uncovered distinct pharmacodynamic patterns—early-, late-, and model-dependent responses—that endpoint assays alone fail to capture. These dynamic profiles offer valuable insights into drug action mechanisms, potentially guiding more precise therapeutic strategies.

To effectively quantify these temporal pharmacodynamic effects, we introduced the RTG-IC_50_ metric. Unlike classical endpoint IC_50_ measures, RTG-IC_50_ integrates multi-day viability data into a single, robust indicator of drug potency. We used isotonic regression for this metric owing to its demonstrated superiority in handling variability (Fujii et al.^23^). Isotonic regression affords greater specificity and resilience than traditional sigmoidal fits, rendering our RTG-IC_50_ a reliable means of accurately characterizing drug responses across diverse models and conditions. The clinical relevance of RTG-IC_50_ extends beyond technical enhancements in assay performance. By capturing the full temporal dynamics of drug response, this metric can identify compounds with a delayed onset of action that might otherwise be discarded when using endpoint assays, thereby rescuing potentially effective therapies. Moreover, the ability to distinguish between early- and late-responding drugs could inform optimal dosing schedules and treatment durations in clinical trials. Within drug-development pipelines, RTG-IC_50_ values could serve as more predictive biomarkers for patient stratification, as they reflect not merely the final drug effect but also the kinetics of response—a parameter increasingly recognized as pivotal for forecasting clinical outcomes.

The extensive characterization of growth dynamics in PDTCs revealed intriguing associations between PDTC proliferation patterns and breast cancer subtypes. While TNBC models often exhibited aggressive growth, a substantial fraction surprisingly displayed negative or stable GRs *in vitro*, indicating notable heterogeneity within TNBC and reinforcing the necessity for individualized pre-clinical testing approaches. Similarly, our platform identified varying some growth patterns among ER+/HER2- and HER2+ models, further emphasizing PDTCs’ potential to recapitulate patient-specific tumor behavior as seen in our previous studies.^3^

Beyond breast cancer, the RTG-based HTDS pipeline is anticipated to be broadly applicable across other tumor types. The core real-time viability assay measures fundamental metabolic activity and has been. Successfully demonstrated in diver cancer cell models^15^ (e.g., lung carcinoma A549 and leukemia K562/THP-1), indicating that its utility is not limited by tissue-of-origin. The homogeneous, add-and-read format of RTG also supports methodological scalability high-throughput screens have integrated similar real-time assays with robust performance and reproducibility.^15^ Moreover, extending RTG to more complex 3D culture systems such as patient-derived organoids (PDOs) represents a promising avenue to increase physiological relevance. Kinetic viability assays are particularly attractive for PDOs because they enable continuous, non-lytic monitoring of live organoid cultures over time,^24^ allowing longitudinal drug response assessment from the same precious sample. Early feasibility studies have already applied luminescence-based real-time viability readouts to organoid models (e.g., mouse gastric tumor organoids and patient-derived colorectal cancer organoids),^15^^,^^24^ supporting the extension of this approach beyond 2D monolayers. Adapting RTG to defined 3D systems does, however, require careful optimization: large organoid structure can hinder uniform reagent penetration and signal detection.^15^ Despite these challenges, ongoing efforts to validate RTG in PDO platforms are highly encouraging.

Despite these advances, several limitations must be acknowledged. First, depletion of the RTG reagent after roughly 6 days constrains continuous measurements over longer intervals, potentially affecting prolonged assays. This temporal restriction may preclude the detection of late-emerging drug-resistance mechanisms or delayed cytotoxic effects that manifest beyond the 6-day window. In addition, the gradual decline in luminescent signal intensity could introduce measurement bias at the later time points, potentially underestimating changes in cell viability between days 5 and 7. Second, the moderate correlation between RTG-derived metrics and conventional endpoint CTG metrics (Pearson’s ρ ≈ 0.49–0.61) indicates that, while RTG captures additional dynamic information, endpoint assays still provide complementary insights into drug effects. This discordance may stem from several confounding factors: (1) the RTG assay measures metabolic activity as a surrogate for viability, which may not perfectly correlate with ATP content quantified by CTG, particularly in cells undergoing metabolic adaptation to treatment; (2) the continuous presence of RTG reagents in the culture medium could interfere with certain drug mechanisms or cellular processes, although we did not observe overt toxicity; and (3) the integration of temporal data in RTG-IC_50_ calculations inherently weights early time points differently from single endpoint measurements, which may explain the moderate correlation.

Furthermore, the experimental design possesses intrinsic limitations that merit consideration. The 384-well format, while enabling high-throughput screening, provides limited culture volume that may affect nutrient availability and waste accumulation over the 7-day assay period. Finally, inter-PDTC variability in GRs remains a challenge, even with our normalization approach, as models with negative GRs may exhibit floor effects that complicate the interpretation of drug responses.

Looking forward, a critical next step involves further validation and refinement of our RTG-IC_50_ metric across broader datasets, including additional tumor types and drug classes. We anticipate such efforts will enhance its generalizability and robustness in pre-clinical drug screening contexts. Moreover, exploring combination therapies using our dynamic monitoring approach might uncover synergistic interactions otherwise undetectable with traditional static assays. Finally, translating these pre-clinical insights into clinical correlates will be instrumental in realizing precision oncology strategies guided by PDTC-based drug screening.

In summary, our integrated RTG-based platform significantly enhances the analytical rigor and biological relevance of HTDS in PDTCs. By capturing nuanced relevance drug-response kinetics through metrics like RTG-IC50, this approach positions PDTC assays as more scalable, and clinically translatable tools in precision cancer medicine.

### Limitations of the study

Despite its advantages, the real-time assay has inherent constraints. First, the luminescent signal is sustainable for only ∼5–6 days before the RTG reagent is depleted, which limits the duration of continuous measurements and may cause late-arising drug effects to be missed. Second, RTG-based viability metrics showed only moderate correlation with traditional ATP-based endpoint assays, indicating that conventional endpoint reads still provide complementary information. This discrepancy likely arises because the RTG assay measures metabolic activity as a surrogate for viability (which can diverge from true ATP content), the continuous presence of reagent might subtly interfere with cellular processes and time-integrated luminescence data weight early responses differently than single time point measures. Finally, variability in PDTC growth dynamics remains a challenge: even with growth-normalization, some models (e.g., those with minimal or negative growth) can exhibit floor effects that complicate the interpretation of drug response metrics.

## Resource availability

### Lead contact

Requests for further information and resources should be directed to and will be fulfilled by the lead contact, Carlos Caldas (cc234@cam.ac.uk).

### Materials availability

The breast cancer PDTX models generated and used in this study are available from the CRUK Cambridge Institute Breast Cancer Functional Genomics Research Group biobank under a material transfer agreement (MTA).

### Data and code availability

All process data (drug response CTG, RTG, and metric) supporting this study have been deposited at Zenodo (https://zenodo.org/records/18633684). All original code has been deposited at Zenodo (https://zenodo.org/records/18633684) and is publicly available as of the date of publication. Any additional information required to reanalyze the raw data reported in this paper is available from the lead contact upon request.

## Acknowledgments

C. Caldas was supported by funding from 10.13039/501100000289CRUK (grant numbers A17197, A27657, and A29580), an NIHR Senior Investigator Award (grant number NF-SI-0515-10090), and a European Research Council Advanced Award (grant number 694620). O.M.R. was supported by the 10.13039/501100018956NIHR Cambridge Biomedical Research Centre (BRC-1215-20014) and the 10.13039/100014013UKRI (UK; MC_UU_0002/16). D.G.-R. is funded by the 10.13039/501100003343Cambridge Commonwealth, European and International Trust. BioRender was used to create some figures under license MX28ZKW91M and VB28ZKWGUB. We are grateful for the generosity of all the patients who donated samples for the development of the tumor xenograft models and the CRUK Cambridge Institute Core Facilities (BRU, Histopathology) for support during the execution of this project.

## Author contributions

C. Caldas and O.M.R. conceived the study; C. Caldas, O.M.R., and D.G.-R. wrote the manuscript; PDTX-derived tumor cells experiments were designed and led by E.E. with input from O.M.R., A.B., A.S., W.G., and D.G.-R.; A.J.W.L. performed validation experiments; Computational and statistical analyses were led by D.G.-R. under the supervision of O.M.R. L.V.N., A.J.W.L., E.E., R.M., P.E., and M.C. provided feedback for the manuscript. C. Crafter, S.F., and M.L. were the pharmaceutical collaborators from AstraZeneca or Varsity and drug defining of PDTCs. All authors read and approved the manuscript.

## Declaration of interests

The authors declare no competing interests.

## STAR★Methods

### Key resources table

**Table:** 

REAGENT or RESOURCE	SOURCE	IDENTIFIER
**Antibodies**		

Anti-mouse CD45 antibody	Abcam	Cat# ab25386; Clone I3/2.3; RRID: AB_470499
Anti-human CD45 antibody (clones 2B11+PD7/26)	Dako (Agilent)	Cat# M0701; Clone 2B11 + PD7/26; RRID: AB_2314143
Anti-human CD20 antibody (clone L26)	Novocastra (Leica Biosystems)	Cat# NCL-L-CD20-L26; Clone L26; RRID: AB_563434
Anti-human AE1/AE3 cytokeratin antibody	Dako (Agilent)	Cat# M3515; Clone AE1/AE3; RRID: AB_2132885

**Biological samples**		

Breast cancer patient-derived tumor xenografts (PDTX)	CRUK Cambridge Institute Breast Cancer Functional Genomics Research Group biobank	N/A
Patient-derived tumor cells (PDTCs)	CRUK Cambridge Institute biobank	N/A

**Chemicals, peptides, and recombinant proteins**		

Dimethyl sulfoxide (DMSO)	Sigma-Aldrich	Cat# D2650; CAS: 67-68-5
Fetal bovine serum (FBS)	Gibco (Thermo Fisher Scientific)	Cat# 10270106
RPMI 1640 Medium	Gibco (Thermo Fisher Scientific)	Cat# 11875093
Recombinant Human EGF	PeproTech (Thermo Fisher Scientific)	Cat# AF-100-15
Recombinant Human FGF-basic (FGF-2/bFGF)	Gibco (Thermo Fisher Scientific)	Cat# PHG0266
B-27 Supplement (50X), serum free	Gibco (Thermo Fisher Scientific)	Cat# 17504044
Penicillin-Streptomycin (10,000 U/mL)	Gibco (Thermo Fisher Scientific)	Cat# 15140122
Gentamicin (50 mg/mL)	Gibco (Thermo Fisher Scientific)	Cat# 15750060

**Critical commercial assays**		

Maxwell RSC Tissue DNA Kit	Promega	Cat# AS1610
CellTiter-Glo 3D Cell Viability Assay	Promega	Cat# G9681
RealTime-Glo MT Cell Viability Assay	Promega	Cat# G9711
Human Tumor Dissociation Kit	Miltenyi Biotec	Cat# 130-095-929

**Deposited data**		

All process data (drug response CTG, RTG and metric)	This paper	Zenodo (https://zenodo.org/records/18633684) and Github (https://github.com/cclab-brca/Assessing_drug_responses_PDTCs)

**Experimental models: Cell lines**		

HCC1937, ER-/HER2-	American Type Culture Collection (ATCC)	CRL-2336, RRID: CVCL_0290

**Software and algorithms**		

R (statistical computing)	R Foundation for Statistical Computing	https://www.r-project.org/; RRID: SCR_001905
flux R package (trapezoidal integration)	CRAN	https://CRAN.R-project.org/package=flux
stats:isoreg (isotonic regression, pooled-adjacent-violators algorithm)	R base (stats package)	https://www.r-project.org/
Original code	This paper	Zenodo (https://zenodo.org/records/18633684)

**Other**		

Maxwell RSC 48 Instrument	Promega	Cat# AS8500
GentleMACS Dissociator	Miltenyi Biotec	Cat# 130-093-235
Falcon 40 μm Cell Strainer	Corning (BD Biosciences)	Cat# 352340
384-well microplates, F-bottom, μCLEAR	Greiner Bio-One	Cat# 781096
PHERAstar FS Plate Reader	BMG LABTECH	CRUK CI
Echo 550 Liquid Handler	Labcyte (Beckman Coulter)	AstraZeneca
Vi-Cell XR Cell Counter	Beckman Coulter	CRUK CI
NucleoCounter NC-200	ChemoMetec	CRUK CI
STR testing service (capillary electrophoresis)	Genetica DNA Laboratories	N/A

### Experimental model and study participant details

#### Breast cancer PDTX samples and specimen validation

Breast Cancer patient-derived tumor xenografts (PDTX) were from the CRUK Cambridge Institute Breast Cancer Functional Genomics Research Group biobank. Their ER/HER2 status was determined using IHC on TMAs of FFPE PDTX tissues (Bruna et al., 2016).

Two methods of Short Tandem Repeat (STR) and Immunohistochemistry (IHC) support the detection of the event of mouse tumor generation and the validation of human tumors.

The DNA extraction from PDTX tissues was performed using Maxwell RSC Tissue DNA kit and Maxwell RSC 48 instrument (Promega) according to the manufacturer instructions. The extracted DNAs were sent to ‘Genetica DNA Laboratories’ to perform the STR test using capillary electrophoresis. Then, the STR negative human samples tested by IHC staining with the antibodies, e.g., CD45 mouse (Abcam, AB25386), CD45 human (Dako, M0701), CD20 human (Novocastra, NCL-L-CD20-L26) and AE1/AE3 human (Dako, M3515).

### Method details

#### Cell viability assays

Single cell suspensions generated from PDTX tissues were cultured (50 mL per well) at different cell concentrations (25000, 50000 and 150000 cells/well, depending on the purpose of the associated experiment) into 384-well plates.

To profile the growth of the cultured PDTCs in 12 days, CellTiterGlo 3D Cell Viability (Promega) was used, according to the manufacturer instructions. The associated signals were measured using the Plate Reader PHERAstar FS. (BMG LABTECH) on days 0, 3, 7, 10 and 12. The test reagent was added to the plates on the endpoint date.

In the drug dose-response experiments, the selected drug was added to wells after 24h of seeding (day0) using Echo Liquid Handler 550 (Labcyte). To quantify drug responses in PDTC, two different cell viability assays were used. In one method, cell viability reading intensities were obtained 6 or 7 days after treatment using the CellTiter-Glo 3D cell viability assay and normalized against blank wells and control wells treated with dimethyl sulfoxide (DMSO, Sigma).

In another method, real-time monitoring of cell viability was performed using a RealTime-Glo MT cell viability assay (Promega). Readingintensities were obtained on days 0, 1, 2, 3, 6 and 7. The substrate and enzyme were added to the cell suspension before seeding in the plates according to the manufacturer’s instructions.

The RealTime-Glo MT Cell Viability assay provided the possibility of monitoring cell viability in every individual well in the course of the experiment, which is not possible while using the cell lytic assay (CellTiter-Glo 3D Cell Viability). This real-time method also supports reducing the amount of PDTCs required for the dose-response real-time study compared with the cell lytic assay.

#### Generation of viable patient-derived tumor cells (PDTCs)

The cryopreserved PDTX tissues were stored in fetal bovine serum (FBS) with 10% DMSO in liquid nitrogen. The frozen tissues were thawed into RPMI (Gibco, Invitrogen) and dissociated into single-cell suspensions by combining mechanical and enzymatic dissociation using the soft tumor dissociation protocol on a GentleMACS Dissociator and the human tumor dissociation kit (Miltenyi) according to the manufacturer instructions. Single-cell suspensions were filtered through 40μm microfilters (BD Biosciences) and transferred into RPMI (Gibco, Invitrogen) which was supplemented using 100μL EGF (Peprotech), 100μL FGF (Invitrogen), 10mL B27 (Thermo Sc.), 5mL PEN STRP (Gibco) and 50μL GENTAMICIN (Gibco). We assessed the cell viability percentage and viable cell concentration comparing two cell counting instruments, Vi-Cell XR Cell Counter (Beckman Coulter) and NucleoCounting NC-200 (Chemometec). As both cell counters show the same cell viability percentage and viable cell concentration, the number of viable cells in the suspensions was assessed using the Vi-Cell XR Cell Counter. Then, cells were cultured in the 384-well plates (Greiner Bio-One) and kept in a humidified cell culture incubator at 37°C with 5% CO2.

### Quantification and statistical analysis

#### Comparison of CTG and RTG assays

The growth rates after 7 days for CTG were calculated as the median intensity of day 7 and the median intensity on day 0 after background normalisation. For RTG, we calculated the ratio of intensities on day 7 and day 0 for each well and took the median of those ratios. The corrected logarithmic scores (adding 0.01 before computing the log) of these ratios were used to compute the Pearson correlation.

#### Statistical analysis of measurement variability

To quantify the variance reduction achieved by same-well monitoring, we calculated coefficient of variation (CV) for each method and time point. Growth differences between Day 7 and Day 3 were calculated differently for each method to account for their distinct viability cell measurement approaches:

For same well and cells RTG monitoring.

• Each well’s growth = (Growth_Day7 – Growth _Day 3)_same_well
• Mean growth across n wells:

$$\bar{y}=\left(\frac{1}{n}\right)\;\sum_{i=1}^{n}\left({\text{Growth}}_{{\text{Day}7}_{{\text{well}}_{i}}}-{\text{Growth}}_{{\text{Day}3}_{{\text{well}}_{i}}}\right)$$

• Growth = Mean(Day7_wells) - Mean(Day3_wells)

CV estimation:

For each Day and each group g ∈ { RTG, CTG}, let the observations be x_1_, …, x_n_.

• Sample mean: ${\hat{\mu }}_{g}=\frac{1}{n}\;\sum_{1}^{n}x_{i}$
• Sample SD (with Bessel’s correction): $s_{g}=\sqrt{\frac{1}{n-1}\;\sum_{1}^{n}{\left(x_{i}-{\hat{\mu }}_{g}\right)}^{2}}$
• Sample variance: $s_{g}^{2}$
• Coefficient of variation (as a percentage):

$${CV}_{g}=100\times \frac{s_{g}}{{\hat{\mu }}_{g}}$$

We compute exactly these quantities per Day: mean_∗, sd_∗, variance_∗, and cv_∗ = (sd_∗/mean_∗)∗100. This variance decomposition demonstrates mathematically why same-well tracking reduces measurement noise by eliminating the well-to-well. Variation component.

#### Growth-rate estimation and categorical classification

To characterise PDTC growth dynamics across seeding densities, we fitted, for each model and experimental replicated (defined by plate and seeding density), a linear regression of log(normalised intensity +0.01) on day to estimate the per-day growth rate (slope), its standard error (SE) and a 95% confidence interval (CI) using the t-distribution (df = *n*-2).Replicate-level slopes were then aggregated to a model-level slope by inverse-variance weighting (weights=1SE2), which provides a precision-aware summary across replicates and densities. Models were classified as positive if the lower 95% CI bound >0, negative if the upper 95% CI bound <0, and neutral otherwise; this CI-based criterion yields an objective, uncertainty-aware threshold instead of an arbitrary cut-off. For descriptive summaries we also report the mean growth rate per model. Robustness of the classification was confirmed across seeding densities, and in a sensitive analysis using a pragmatic fixed boundary (±0.08 log-intensity/day), which showed high concordance with the CI-based rule for all the PDTCs with extended time of 12 days (Figure S1A, see code).

#### Computation of AUCs and IC50 (replicates, missing values, isotonic regression)

For each plate and day, raw luminescence was blank-corrected using the median of blank wells, then truncated to [0,DMSO-Blank]. Signals were expressed as percent inhibition relative to the day-model-input-matched DMSO median and truncated to [0.100]. For each dose we had three technical replicates (wells). Within each replicate we computed the area under the time course by trapezoidal integration (R package flux), after excluding non-finite points and sorting by time; replicates series with <2 time points after filtering were discarded. Replicate AUCs greater than the corresponding DMSO AUC were capped at the DMSO AUC to avoid negative inhibitions, and converted to percent inhibition as

$$\%\;\text{inhibition}=100\times \left(1-\frac{{\text{AUC}}_{\text{rep}}}{{\text{AUC}}_{\text{DMSO}}}\right)$$

Per-dose inhibition was then summarised by the mean across valid replicates (and the SD for display bands).

Dose–response curves were estimated by nonparametric isotonic regression (statsisoreg, pooled–adjacent–violators algorithm) applied to the vector of per-dose mean inhibitions (one value per dose). Because isotonic regression is order-based, fitting on dose or on *log*_10_ (dose) yields the same estimator; we plot *log*_10_ (dose) for readability and interpretability. To visualise uncertainty, we computed display bands by applying isoreg to mean ±1.96 · SD; these are descriptive bands. IC50 was defined as the *log*_10_ (dose) at which the isotonized mean curve crossed 50% inhibition, obtained by linear interpolation on a dense *log*_10_ (dose) grid; IC50 was reported as not estimable when 50% inhibition was not reached. Prior to all isoreg calls, non-finite x or yvalues were dropped to meet the function’s requirement for finite inputs. As a robustness check, we verified that fitting isoreg directly to all replicate inhibition values (with tied x at each dose, which isoreg averages internally) produced equivalent qualitative results.

#### CTG and RTG heatmap

Delta AUC was defined as the absolute value of the difference between the AUC computed from the Day 7 CTG-based drug-response assay and the AUC computed from the RTG-based assay on the same day.

#### Dose-response fitting and display

For RTG and CTG AUC-based analysis we fit isotonic dose-response curves (monotone-increasing in $-inhibition, equivalently monotone-decreasing in viability). When the empirical response is flat or increases with dose (e.g., partial agonism/pro-proliferative effects), the isotonic curve cannot cross the 50% threshold and RTG-IC_50_ is reported as not estimable, NA or “>highest tested dose”. For visualisation, we provide per-assay plots for all screens (RTG *n* = 2604, CTG *n* = 2520, radiotherapy CTG *n* = 240; radiotherapy *n* = 144); CTG plots additionally include M-spline overlays as an alternative smooth representation.^25^

## Supplemental information

Document S1. Figures S1–S7

Document S2. Article plus supplemental information
