# Supplementary material for: Real-time cell viability monitoring for high-throughput drug screening using tumor xenograft-derived cells
Source: Cell Rep Methods. 2026 May 27;6(7):101464. doi: 10.1016/j.crmeth.2026.101464 (PMC13389979; doi:10.1016/j.crmeth.2026.101464)
Supplement: Document S1. Figures S1–S7 [file mmc1.pdf]

Cell Reports Methods, Volume *6*

Supplemental information

Real-time cell viability monitoring

for high-throughput drug

screening using tumor xenograft-derived cells

Elham Esmaeilishirazifard, Daniel Guerrero-Romero, Allan J.W. Lui, Abigail Shea, Long V. Nguyen, Maurizio Callari, Riccardo Masina, Kevin Tu, Richard Baird, Paul Edwards, Wendy Greenwood, Steve Fuller, Claire Crafter, Mandy Lawson, Alejandra Bruna, Oscar M. Rueda, and Carlos Caldas

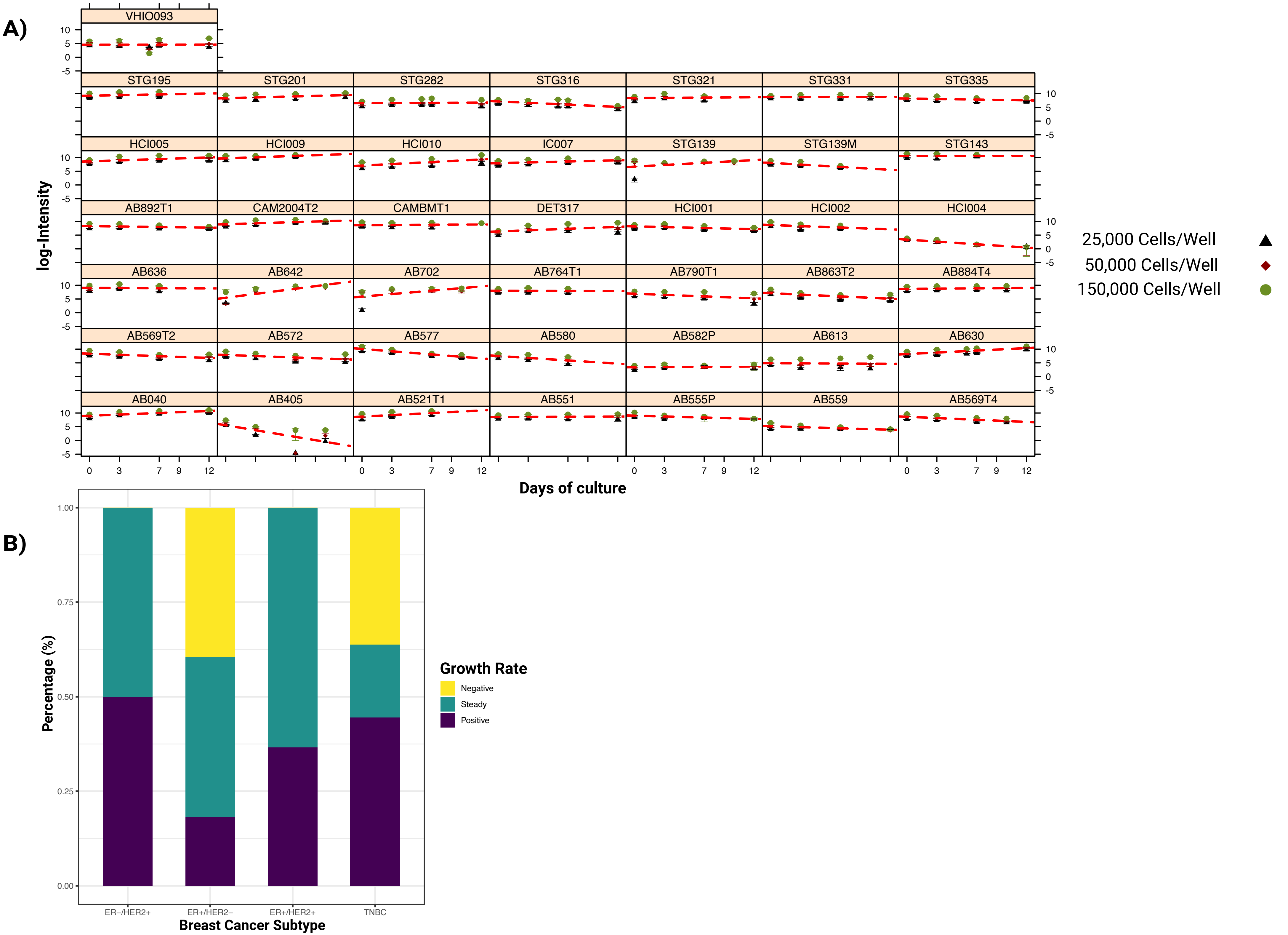
A)
VHIO093
STG195
STG201
STG282
STG316
STG321
STG331
STG335
HCI005
HCI009
HCI010
IC007
STG139
STG139M
STG143
AB892T1
CAM2004T2
CAMBMT1
DET317
HCI001
HCI002
HCI004
AB636
AB642
AB702
AB764T1
AB790T1
AB863T2
AB884T4
AB569T2
AB572
AB577
AB580
AB582P
AB613
AB630
AB040
AB405
AB521T1
AB551
AB555P
AB559
AB569T4
log-Intensity
Days of culture
25,000 Cells/Well
50,000 Cells/Well
150,000 Cells/Well
B)
Percentage (%)
Breast Cancer Subtype
ER−/HER2+
ER+/HER2−
ER+/HER2+
TNBC
Growth Rate
Negative
Steady
Positive

Supplementary Figure 1: Extended growth profiles of PDTCs (related to Figure 2).

(A) Plots showing normalised luminescence intensity of the 43 PDTCs at different time points using the CellTiter-Glo 3D cell viability read-out during a 12-day experiment. Colours indicate three initial seeding cell densities (25,000, 50,000 and 150,000 cells/well).

(B) Distribution of PDTCs' growth rates across different breast cancer subtypes: ER+/HER2+, ER+/HER2-, ER-/HER2+ and TNBC. The bar plot shows the breast cancer subtypes stratified by growth rate categories: Positive, Steady, and Negative.

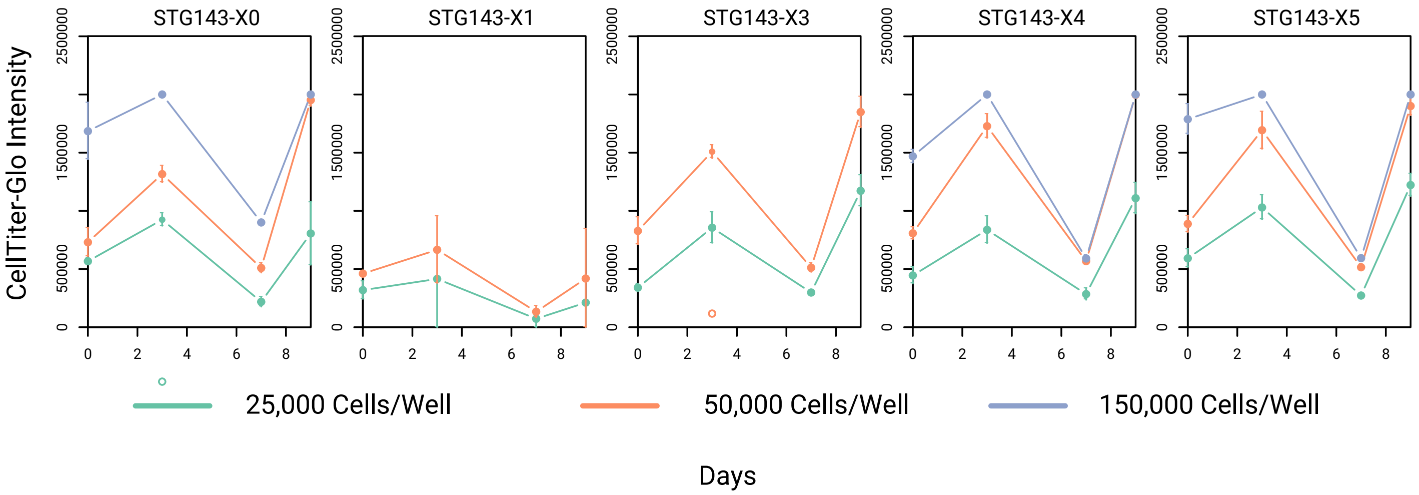

CellTiter-Glo Intensity
STG143-X0
STG143-X1
STG143-X3
STG143-X4
STG143-X5
0
500000
1500000
2500000
0
2
4
6
8
25,000 Cells/Well
50,000 Cells/Well
150,000 Cells/Well
Days

Supplementary Figure 2: Passage consistency in a single PDTC model (related to Figure 2).

Severalpassages (X0, X1, X3, X4 and X5) ofSTG143 PDTX(ER+/HER2-)were tested in three cell densities (25,000, 50,000 and 150,000 cells/well) using the CellTiter-Glo 3D cell viability assay on days 0, 3, 7 and 9. The measurements from each day where obtained from a single plate containing the cells from all the passages.

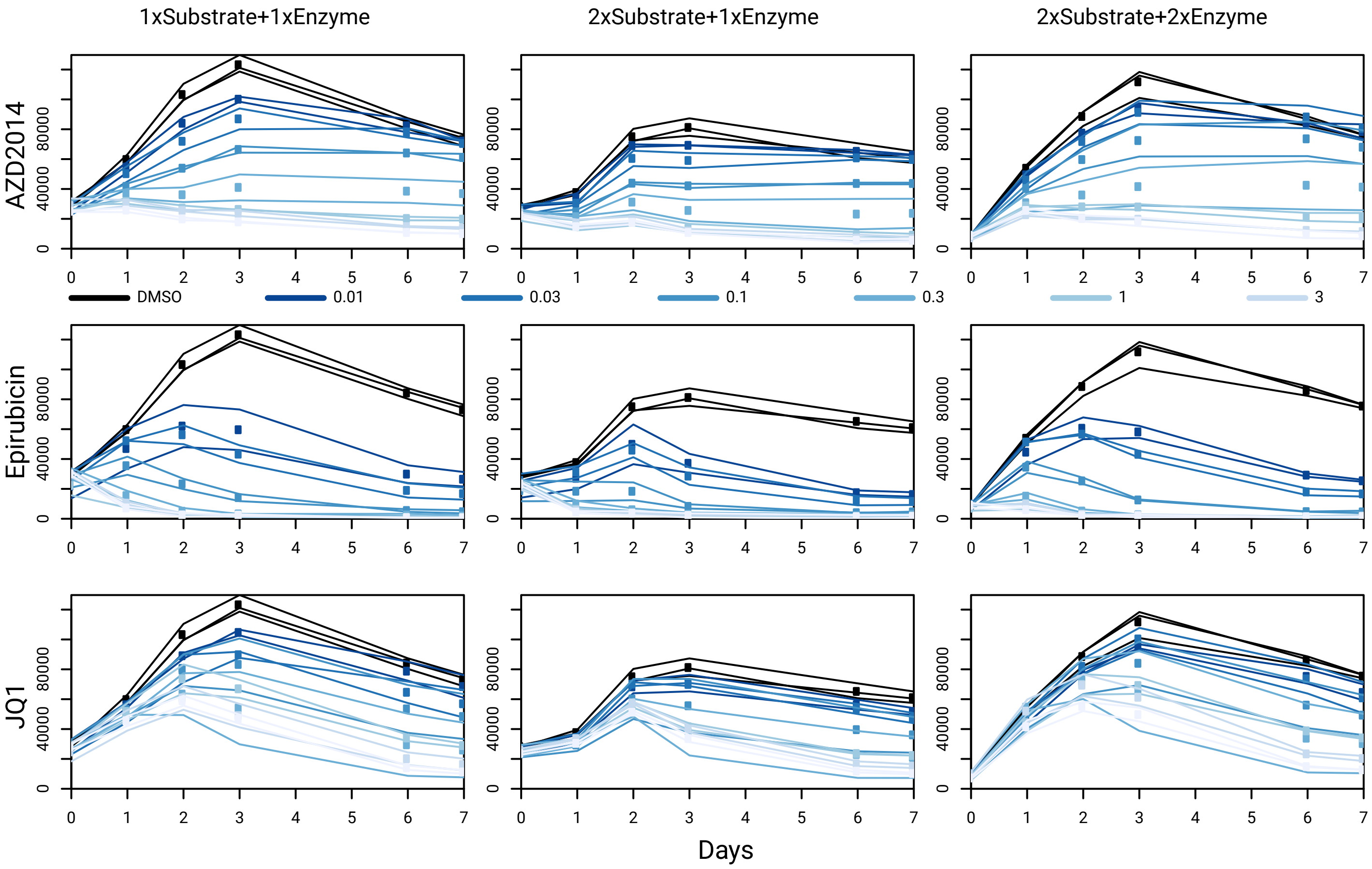

1xSubstrate+1xEnzyme
2xSubstrate+1xEnzyme
2xSubstrate+2xEnzyme
AZD2014
Epirubicin
JQ1
0
40000
80000
0 1 2 3 4 5 6 7
DMSO
0.01
0.03
0.1
0.3
1
3
Days

Supplementary Figure 3: Drug effect on AB521T1 PDTCs using RTG across different substrate-to-enzyme ratios (related to Figure 3).

Evaluation of pharmacological effects of AZD2014, Epirubicin, and JQ1 on the PDTC model AB521T1 under different conditions of RTG assay reagents. Each drug was applied at seven distinct concentrations, represented by varying shades of blue. Control treatment with DMSO is depicted by black lines. The figure is divided into three panels to illustrate the outcomes under different substrate-to-enzyme ratios: 1xSubstrate:1xEnzyme (left panel), 2xSubstrate:1xEnzyme (middle panel), and 2xSubstrate:2xEnzyme (right panel).

A)

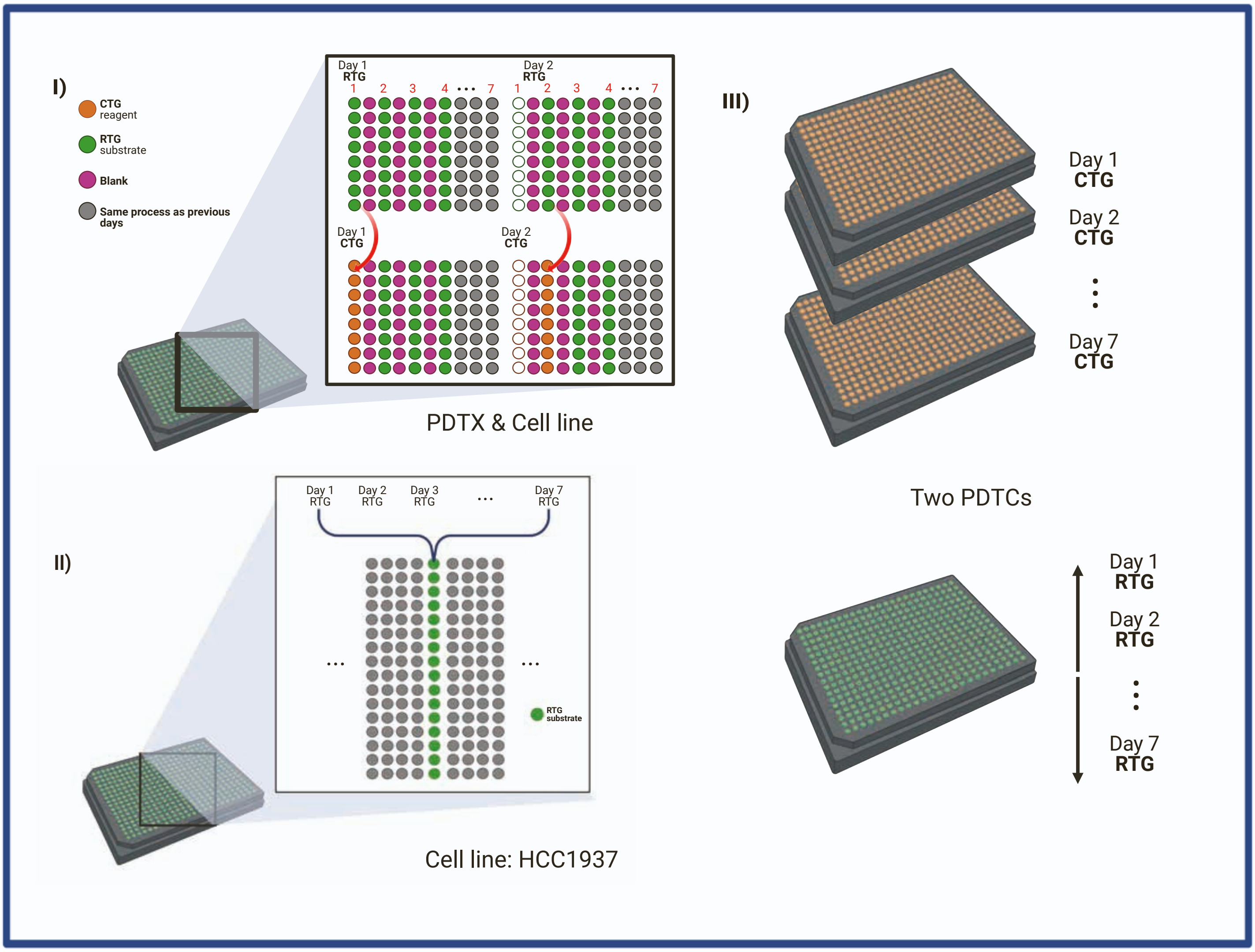
I)
CTG reagent
RTG substrate
Blank
Same process as previous days
Day 1 RTG
Day 2 RTG
Day 1 CTG
Day 2 CTG
PDTX & Cell line
II)
Day 1 RTG
Day 2 RTG
Day 3 RTG
Day 7 RTG
RTG substrate
Cell line: HCC1937
III)
Day 1 CTG
Day 2 CTG
Day 7 CTG
Two PDTCs
Day 1 RTG
Day 2 RTG
Day 7 RTG

B)

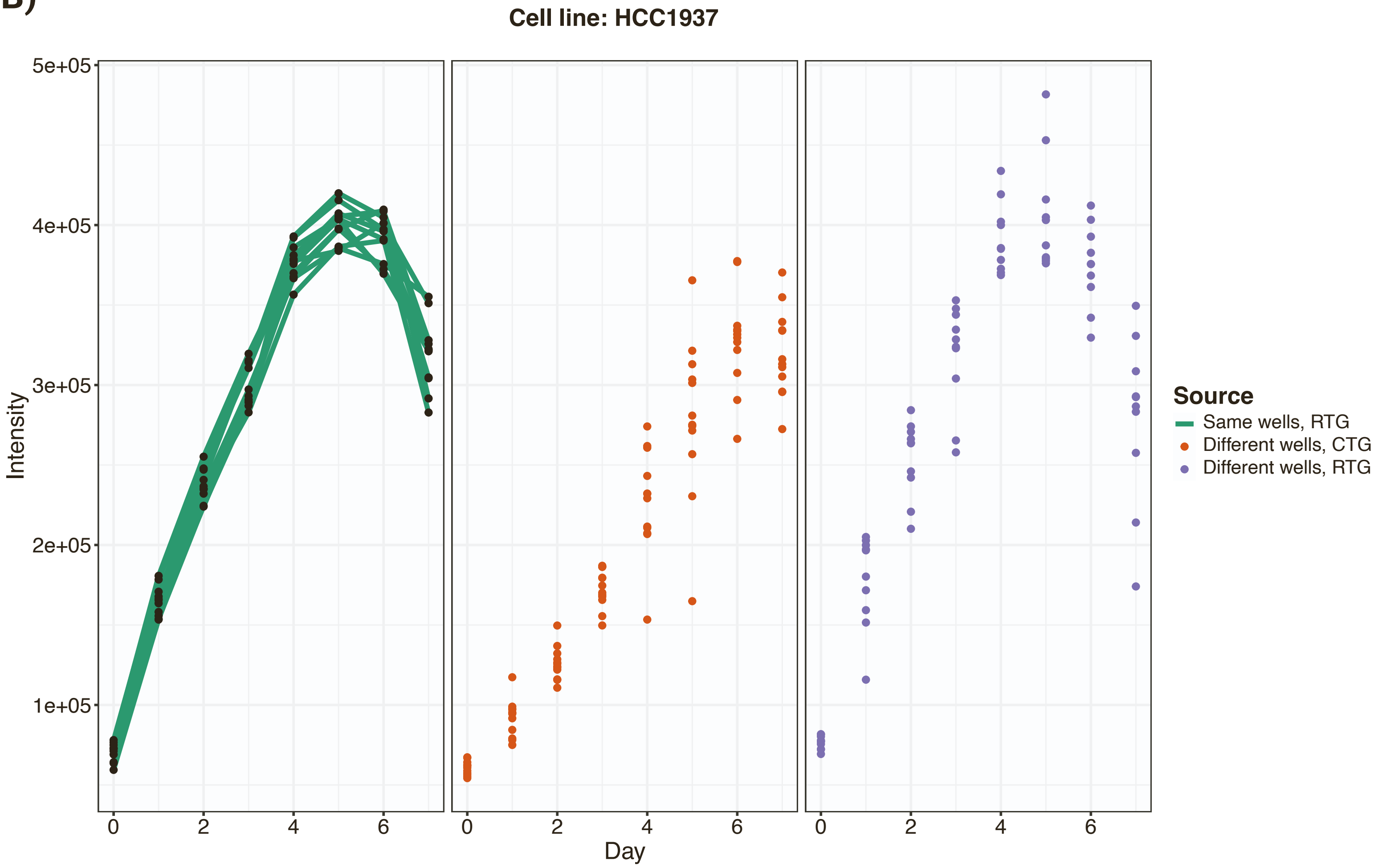
Cell line: HCC1937
Intensity
5e+05
4e+05
3e+05
2e+05
1e+05
0 2 4 6
Day
Source
Same wells, RTG
Different wells, CTG
Different wells, RTG

Supplementary Figure 4: Experimental settings to compare CTG and RTG assay performance (related to Figure 3).

(A) Outline of the three approaches taken to evaluate the results from CTG and RTG on similar HCC1937 cell populations: I) HCC1937 cells were cultured on one plate for the whole duration of the experiment; the same well was subjected to RTG bioluminescent analysis followed by CTG assay (i.e., cell lysis and bioluminescent measurement). One column of the plate was used for each consecutive day. II) HCC1937 cells were seeded in one plate and cell viability was monitored over seven days in each individual well. III) HCC1937 cells were seeded in several plates at the same concentrations. Cells from one plate were used to measure cell viability on one day and then discarded regardless of the use of CTG or RTG. Each plate was only used for one assay and one day, over a period of seven days.

(B) Intensity measurements obtained from the experimental settings shown in (A) for CTG and RTG. Each individual line represents one individual well across days (left panel, experimental setting II), or wells from the same row at different days (middle and right panel, experimental setting I: each well is used as an end-point measurement for its respective day).

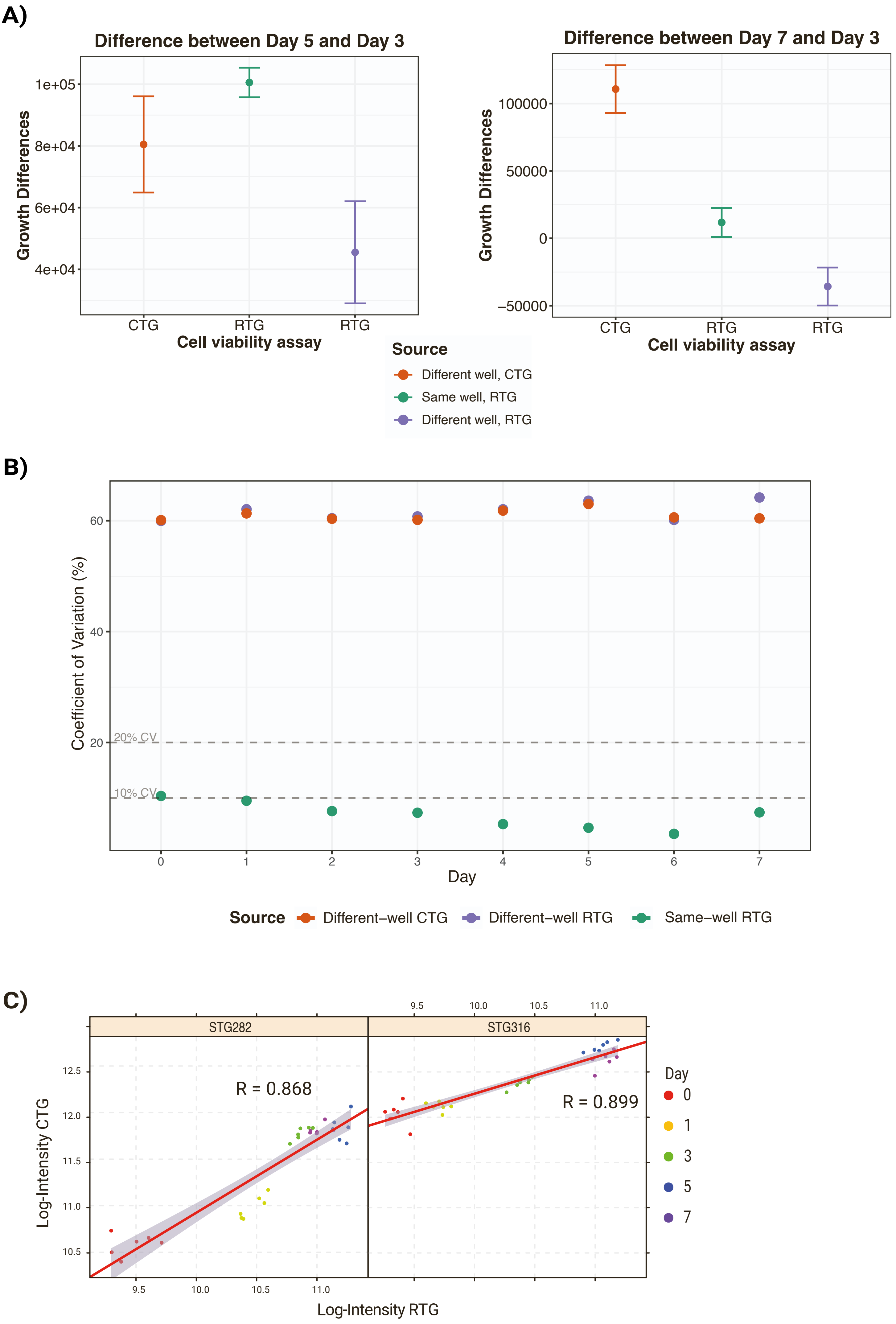
A)
Difference between Day 5 and Day 3
Growth Differences
1e+05
8e+04
6e+04
4e+04
CTG
RTG
RTG
Cell viability assay
Difference between Day 7 and Day 3
Growth Differences
100000
50000
0
−50000
CTG
RTG
RTG
Cell viability assay
Source
Different well, CTG
Same well, RTG
Different well, RTG
B)
Coefficient of Variation (%)
60
40
20
20% CV
10% CV
0
1
2
3
4
5
6
7
Day
Source
Different−well CTG
Different−well RTG
Same−well RTG
C)
STG282
STG316
R = 0.868
R = 0.899
Log-Intensity CTG
12.5
12.0
11.5
11.0
10.5
9.5
10.0
10.5
11.0
Log-Intensity RTG
Day
0
1
3
5
7

Supplementary Figure 5: RTG assay performance on HCC1937 breast cancer cell line (related to Figure 3).

(A) Comparison of the growth between day 5 (and 7) and day 3 for CTG-different wells (setting I), RTG-same wells (setting II), and RTG-different wells (setting I).

(B) Coefficient of variation analysis comparing measurement reproducibility across assays. RTG (green) maintains CV < 10% (lower dashed line, pharmaceutical standard), while different-well CTG (orange) and different-well RTG (blue) both exhibit ~60% CV. Upper dashed line indicates 20% CV threshold. n=16 wells per method per time-point.

(C) Comparison of CTG and RTG in STG282 and STG316 PDTCs from different wells and plates (setting III). Linear regression (red line) estimated using the medians of cell viability from CTG and RTG across different days.

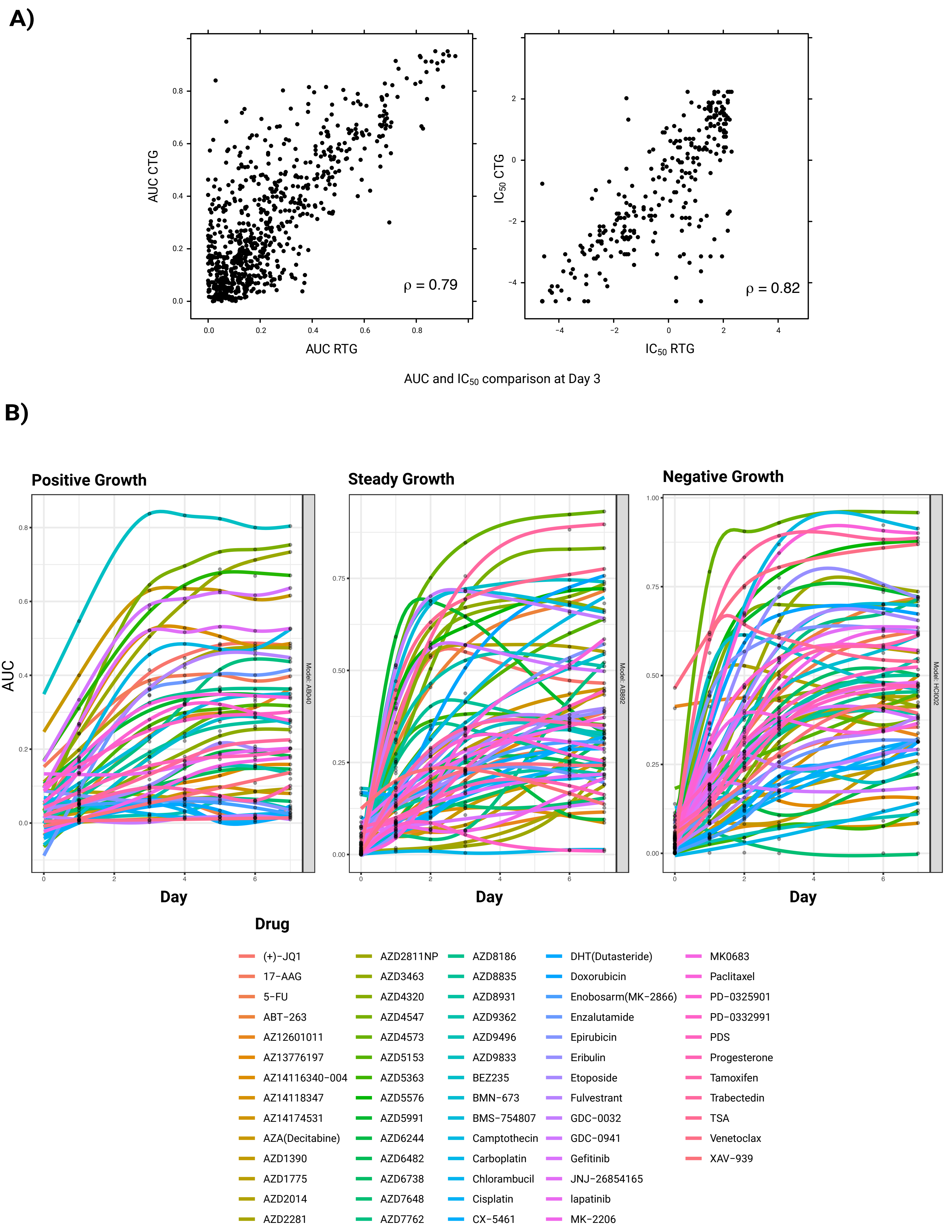
A)
AUC CTG
AUC RTG
ρ = 0.79
IC50 CTG
IC50 RTG
ρ = 0.82
AUC and IC50 comparison at Day 3
B)
Positive Growth
Steady Growth
Negative Growth
AUC
Day
Model: AB040
Model: AB892
Model: HCI002
Drug
(+)−JQ1
17−AAG
5−FU
ABT−263
AZ12601011
AZ13776197
AZ14116340−004
AZ14118347
AZ14174531
AZA(Decitabine)
AZD1390
AZD1775
AZD2014
AZD2281
AZD2811NP
AZD3463
AZD4320
AZD4547
AZD4573
AZD5153
AZD5363
AZD5576
AZD5991
AZD6244
AZD6482
AZD6738
AZD7648
AZD7762
AZD8186
AZD8835
AZD8931
AZD9362
AZD9496
AZD9833
BEZ235
BMN−673
BMS−754807
Camptothecin
Carboplatin
Chlorambucil
Cisplatin
CX−5461
DHT(Dutasteride)
Doxorubicin
Enobosarm(MK−2866)
Enzalutamide
Epirubicin
Eribulin
Etoposide
Fulvestrant
GDC−0032
GDC−0941
Gefitinib
JNJ−26854165
lapatinib
MK−2206
MK0683
Paclitaxel
PD−0325901
PD−0332991
PDS
Progesterone
Tamoxifen
Trabectedin
TSA
Venetoclax
XAV−939

Supplementary Figure 6: PDTC pharmacokinetics are PDTC-model dependent (related to Figure 4).

(A) Comparison of AUC scores and IC_{50} obtained with CTG and RTG on day 3, from 12 PDTC models and 67 drugs show a total of 1,081 AUC scores.

(B) Examples of B-spline regression (performed on RTG-AUC values up to day 7) from three PDTC models displaying positive (AB040, left panel), steady (AB892, middle panel), and negative growth (HCI002, right panel). Each line indicates the AUC obtained from one compound (of a total of 67 drugs).

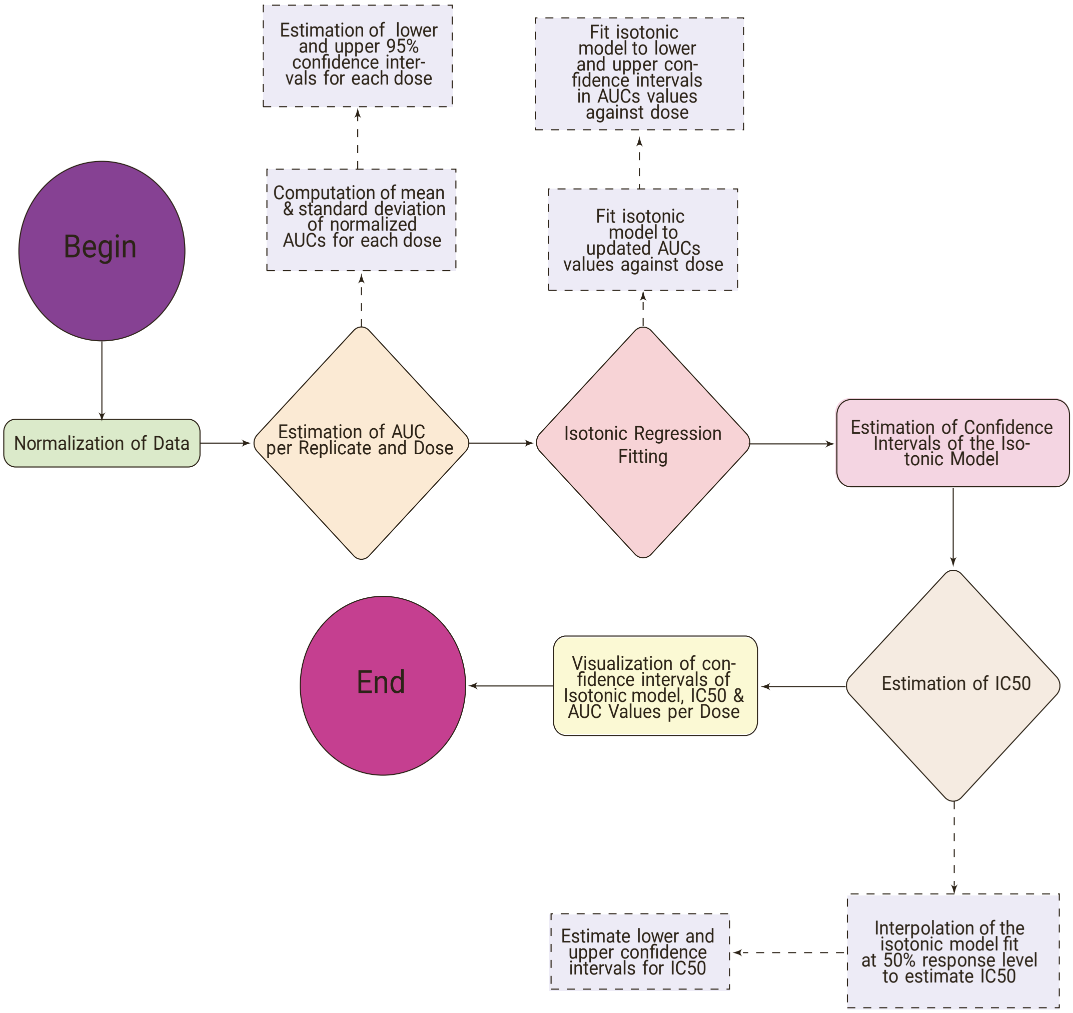
Begin
Normalization of Data
Estimation of AUC per Replicate and Dose
Computation of mean & standard deviation of normalized AUCs for each dose
Estimation of lower and upper 95% confidence intervals for each dose
Isotonic Regression Fitting
Fit isotonic model to updated AUCs values against dose
Fit isotonic model to lower and upper confidence intervals in AUCs values against dose
Estimation of Confidence Intervals of the Isotonic Model
Estimation of IC50
Interpolation of the isotonic model fit at 50% response level to estimate IC50
Estimate lower and upper confidence intervals for IC50
Visualization of confidence intervals of Isotonic model, IC50 & AUC Values per Dose
End

Supplementary Figure 7: Application of the RTG drug response metric (related to Figure 5).

Schematic flowchart detailing the algorithm followed to implement the RTG drug response metric.
